# Long-Term Effects of Human Papillomavirus Vaccination in Clinical Trials and Real-World Data: A Systematic Review

**DOI:** 10.3390/vaccines10020256

**Published:** 2022-02-08

**Authors:** Megumi Kurosawa, Masayuki Sekine, Manako Yamaguchi, Risa Kudo, Sharon J. B. Hanley, Megumi Hara, Sosuke Adachi, Yutaka Ueda, Etsuko Miyagi, Sayaka Ikeda, Asami Yagi, Takayuki Enomoto

**Affiliations:** 1 Department of Obstetrics and Gynecology, Niigata University Graduate School of Medical and Dental Sciences, Niigata 951-8520, Japan; m-kurosawa@med.niigata-u.ac.jp (M.K.); manako0131@med.niigata-u.ac.jp (M.Y.); pearpear@med.niigata-u.ac.jp (R.K.); sadachi@med.niigata-u.ac.jp (S.A.); enomoto@med.niigata-u.ac.jp (T.E.); 2 Center for Environmental and Health Sciences, Hokkaido University, Sapporo 060-8638, Japan; sjbh1810@med.hokudai.ac.jp; 3 Department of Preventive Medicine, Faculty of Medicine, Saga University, Saga 849-8501, Japan; harameg@cc.saga-u.ac.jp; 4 Departments of Obstetrics and Gynecology, Osaka University Graduate School of Medicine, Osaka 565-0871, Japan; y.ueda@gyne.med.osaka-u.ac.jp (Y.U.); a.yagi@gyne.med.osaka-u.ac.jp (A.Y.); 5 Department of Obstetrics and Gynecology, Yokohama City University School of Medicine, Yokohama 236-0004, Japan; emiyagi@yokohama-cu.ac.jp; 6 Division of Surveillance and Policy Evaluation, Institute for Cancer Control, National Cancer Center, Tokyo 104-0045, Japan; sayakaikeda0201@gmail.com

**Keywords:** HPV vaccination, long-term effect, clinical trial, real-world data, seropositivity

## Abstract

The preventive effect of HPV vaccines against anogenital and oropharyngeal cancers has been proven in both clinical trials and real-world data. We reviewed the published evidence about the long-term efficacy and effectiveness of the HPV vaccine in available papers of clinical trials and real-world data. As far as we searched, the longest period of preventive effect for the bivalent, 4-valent, and 9-valent vaccine were 11 years in the Costa Rica trial, 14 years in the FUTURE II, and 8 years in the LTFU extension study of V503-002 and the Scandinavian study, respectively. The sustained clinical effect during the observation period was longest for the 4-valent vaccine. In real-world data, the longest observation period of the vaccine effectiveness was 12 years in an Australian study for the 4-valent vaccine. On the other hand, the longest period of long-term persistence of HPV vaccine-induced seropositivity was 14 years in FUTURE II for the 4-valent vaccine. For the bivalent vaccine, additional long-term follow-up studies may not have been planned due to the launch of the 4-valent and 9-valent vaccines. In some studies of the 9-valent vaccine, the results have not yet been published because of the short observation period. The additional results are expected in the future. In a national immunization program, most girls and boys are inoculated with HPV vaccine by the time puberty begins; thus, it is important to monitor the vaccine effect at least until the sexually active period in their 20s and 30s.

## 1. Introduction

Human papillomavirus (HPV) is mainly transmitted through sexual activity. In fact, HPV sequences have also been identified in chorionic villi tissues from pregnant females and other districts of the reproductive tract [1,2]. HPV infection is the most common viral infection of the genital tract and persistent infection with high-risk HPV types (HR-HPV) can cause changes from normal cells to precancerous and cancerous lesions [3] in both men and women. It is estimated that about 80% of men and women become infected during their lifetime, and are usually cured by the host’s immune system [4]. Due to persistent HPV infection, HPV DNA is integrated into the host DNA. As a result, the onco-proteins E6 and E7 are expressed, causing the degradation of p53 and pRb, respectively. This is followed by the alteration of many cellular processes such as DNA repair, angiogenesis, apoptosis, etc., which ultimately lead to carcinogenesis [4].

More than 200 types of HPV have been identified, which are classified into high-risk types (such as types 16, 18, 31, 33, 35, 39, 45, 51, 52, 56, 58, 59, 66, 68, 73, and 82) that are carcinogenic, and low-risk types (such as types 6, 11, 42, 43, and 44) that cause anogenital warts and benign tumors, such as condyloma acuminata. High-risk HPV types are thought to be responsible for 7–8% of human malignancies, including 96% of cervical cancers, 93% of anal cancers, 64% of vaginal cancers, 51% of vulvar cancers, 36% of penile cancers, and 63% of oropharyngeal cancers. Among them, the risk of carcinogenesis from type 16 and 18 infections is particularly high [5].

HPV is estimated to cause nearly 36,000 cases of cancer in men and women every year in the United States. HPV vaccination can prevent 33,000 of these cancers by preventing the infections that cause them [6]. Especially in the prevention of cervical cancer, many reports from clinical trials have shown that the preventive effect of HPV16/18 infection and the development of cervical precancerous lesions are close to 100% [7,8,9,10,11,12,13,14,15,16]. The preventive effect against invasive cervical cancer has also been clearly proven worldwide. A significant reduction in invasive cervical cancer was reported in Sweden in 2020, followed by Denmark and England in 2021 in real-world data [17,18,19].

The HPV vaccine has been available since 2006 and targets mainly adolescent girls [20]. Currently, a national immunization program with the HPV vaccine is conducted in more than 100 countries [21]., and most programs target mainly young adolescent girls [22]. However, since the risk of HPV infection persists throughout the period of sexual activity in women, it is important to confirm the long-term preventive effect of the HPV vaccine [23].

## 2. Methods

Three databases (PubMed, Google Scholar, and ClinicalTrials.gov) and references or related articles were used to review the efficacy and effectiveness of HPV vaccination. In the database, related articles were identified using the following search string: (“human papillomavirus vaccination” OR “HPV vaccination” OR “human papillomavirus vaccine” OR “HPV vaccine”) AND (“efficacy” OR “effectiveness” OR “effect” OR “clinical trial”). Given the search results, we added the terms “long term”, “follow up”, “extension”, and “antibody” to cover all relevant articles.

**Figure 1:**
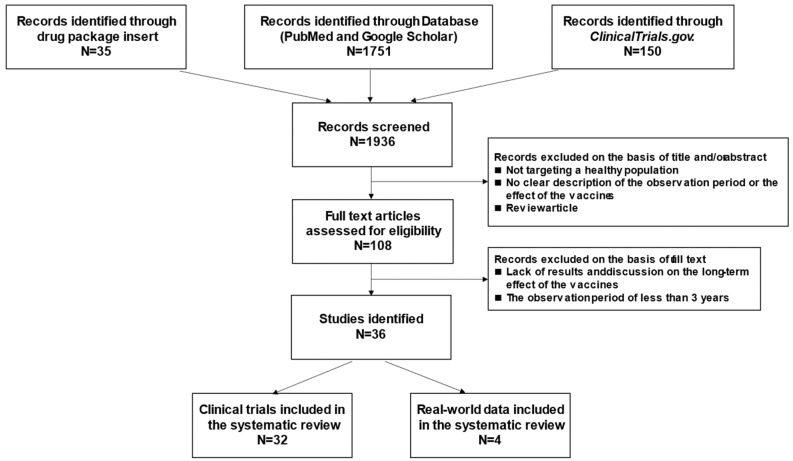
Flow-chart diagram of study selection. PubMed and Google Scholar literature searches yielded a total of 1751 records. In addition, we found 35 literatures listed in the package insert and 150 studies in ClinicalTrials.gov of the HPV vaccines. We screened 1936 records at the abstract/title level for eligibility according to exclusion criteria. Finally, 32 clinical trials and 4 observational studies were included for this review.

PubMed and Google Scholar literature searches yielded a total of 1751 records. In addition, we found 35 literatures listed in the package insert and 150 studies in ClinicalTrials.gov of the HPV vaccines. After that, we screened 1936 records at the abstract/title level for eligibility according to exclusion criteria. Subsequently, 108 records were screened in full-text. A total of 36 publications reported relevant data of long-term effects of HPV vaccination. For this review, 32 clinical trials and 4 observational studies were included (Figure 1).

## 3. Available Vaccines

Currently, three types of HPV vaccines are used worldwide to prevent the development of cervical cancer. The bivalent vaccine (Cervarix^®^) against HPV 16/18 (high-risk HPV type: HR-HPV), 4-valent vaccine (Gardasil^®^) against HPV 6/11 (low-risk HPV type: LR-HPV) and HPV 16/18 (HR-HPV), and 9-valent vaccine (Gardasil 9^®^) against HPV 6/11 (LR-HPV), and HPV 16/18/31/33/45/52 (HR-HPV) [24]. Cervarix^®^ and Gardasil^®^ could prevent 70% of cervical cancer cases, and Gardasil-9^®^ almost 90% [24]. Table 1 shows the vaccination schedule, dose, and adjuvant in each vaccine [25,26,27].

## 4. Long-Term Efficacy: Clinical Trials

Numerous clinical trials have been conducted to date for each vaccine. We considered the difference in efficacy depending on the vaccine used and target age (Table 2, Table 3 and Table 4).

### 4.1. Bivalent Vaccine

#### 4.1.1. Young Women

According to a previous review on the long-term effect on precancers [23], the observation period for the bivalent HPV vaccine was 3.6 years in the HPV 001 [25], 6.4 years in the HPV 007 [34], 8.4 years in the HPV 023 [56], and 9.4 years in the Extension HPV023 trial [34]. In the current situation, the longest follow-up period for bivalent vaccines to date is 11.1 years in the Costa Rica Vaccine Trial [36]. Extension NCT00196937 [37] and NCT 00947115 [35] were clinical trials of evaluations for long-term immunogenicity.

The extension HPV023 trial on a bivalent vaccine was an initial double-blind, randomized, placebo-controlled study in women aged 15–25 years with normal pre-vaccination cytology [34]. As a result, no HPV16/18-associated infections or cyto-histopathological abnormalities occurred in the vaccine group 9.4 years after vaccination. The results showed that no HPV16/18-related infections or pathological abnormalities occurred 9.4 years after vaccination in the vaccine group. Vaccine efficacy (VE) was 100% against HPV-16/18 infection, 95.0% against low grade squamous intraepithelial lesion (LSIL)+, 100% against cervical intraepithelial neoplasia (CIN)1+, and 100% against CIN2+ associated with HPV16/18.

In the Costa Rica Vaccine Trial, a randomized, double-blind, controlled trial that assessed the efficacy of the initial bivalent vaccination, women aged 18–25 years were enrolled. As a result, cumulative VE against HPV 16/18-associated CIN2+ (CIN2 or worse) and CIN3+ (CIN3 or worse) over the 11.1-year period was 97.4% (95% confidence interval [CI], 88.0–99.6) and 94.9% (73.7–99.4), respectively [36]. This report shows the longest efficacy period of a bivalent vaccine.

#### 4.1.2. Adult Women

The longest follow-up period so far was 7 years in the VIVIANE study. In the VIVIANE study, a 7-year follow-up of a double-blind, randomized controlled study involving women age > 25 years; women were randomly assigned (1:1) to receive a bivalent vaccine or aluminum hydroxide control, using an internet-based system. In addition, 4407 women were assigned to a cohort following the protocol for efficacy (vaccinated, n = 2209; controls, *n* = 2198) and 5747 women were assigned to the total vaccination cohort (vaccinated, *n* = 2877; controls, *n* = 2870). The vaccine showed efficacy in preventing persistent HPV16/18 infection, cytological abnormalities, and CIN1+ [32].

#### 4.1.3. Men

The P011-NCT00309166 trial was the first to investigate the efficacy of the bivalent vaccine in boys [37]. In this study, healthy boys aged 10–18 years were randomized (2:1 ratio) to receive the vaccine (*n* = 181) or hepatitis B virus (HBV) control vaccine (*n* = 89) at 0, 1, and 6 months, and were followed for 7 months. The antibody titers of HPV 16/18 in men aged 10–18 and 10–14 years at 7 months after vaccination were higher than those in women aged 15–25 and 10–14 years in previous reports [57].

### 4.2. 4-Valent Vaccine

#### 4.2.1. Young Women

The observation period was 3.6 years in the FUTURE I [39], 5 years in the HPV-P007 [39], 8 years in the Nordic P015 [43], 10 years in the V501-018-11 [44], and 14 years in the FUTURE II [45].

In the V501-018-11 trial on men and women aged 9–15 years, during the 10-year follow-up period no genital warts, cervical and/or genital pre-cancers or cancers were observed [44].

Recently, a final report of the FUTURE II on a 4-valent vaccine was published [45]. In the randomized, double-blind, placebo-controlled study in Northern Europe, women aged 16–23 years received three doses of the 4-valent vaccine and were followed up for an additional ≥ 10 years. No cases of high-grade cervical dysplasia associated with HPV16/18 were observed in the protocol validated population (*n* = 2121) during the entire study period. VE of 100% (95% CI, 94.7–100) was demonstrated at 12 years or more, with a trend toward sustained protection up to 14 years after vaccination.

#### 4.2.2. Adult Women

The longest follow-up period so far was 10 years in the P019-21 trial [26]. The P019 trial evaluated the efficacy of the 4-valent vaccine in women aged 24-45 years [58]. In this study, 3919 women aged 24-45 years, with no history of cervical disease or genital warts in the past 5 years, were enrolled. The 4-valent vaccine or placebo was given on day 1 and, after 2 and 6 months. This study showed the efficacy of the 4-valent vaccine in the prevention of HPV 6/11 (LR-HPV) and HPV 16/18 (HR-HPV) infection, CIN, and external genital lesions (EGL) related to HPV 6/11/16/18 for 4 years. In the Extension P019 and the P019-21 trials, there were no cases of HPV 6/11/16/18-related CIN, AIS and EGL during the 6-10 year extended follow-up [26,42].

#### 4.2.3. Men

Several extension studies of V501-P020 [59,60] were reported previously (P020-AIN substudy [46], NCT00090285 [59] and P020-21 [48]). The longest follow-up period was 10 years (up to 11.5 years) in P020-21 [26,48].

NCT00090285 trial [47] is an open-label, long-term follow-up extension multicentre study conducted at 46 centers in 16 countries. After the P020 and P020-11 trial [46,59], participants who had received at least one dose of the 4-valent HPV vaccine in the previous study were defined as the early vaccination group. Placebo recipients in the previous trial were offered three doses of 4-valent HPV vaccine at the end of the trial, and those who received one or more doses were enrolled in the long-term follow-up (LTFU) trial as the catch-up vaccination group. The early vaccination group was followed for a median of 9.5 years (range, 0.1–11.5) after receiving the third dose of the vaccine, and the catch-up vaccination group was followed for a median of 4.7 years (0.0–6.6) after receiving the third dose. In both groups, there were no new reported cases of HPV 6/11 (LR-HPV) and HPV 16/18 (HR-HPV)-related EGL and there was a lower incidence of HPV 6/11/16/18-related anal intraepithelial neoplasia and anal cancer.

In P020-21, a follow-up study of V501-P020, a preventive effect against HPV 6/11/16/18-related anal cancer and its precursor lesion, condyloma acuminata, was observed for up to 11.5 years after the third inoculation [48]. The V501-P020 trial, a randomized, placebo-controlled, double-blind trial, examined the efficacy of the 4-valent vaccine in preventing infection and genital disease in men [59,60]. In the trial, 4065 healthy men aged 16–26 years from 18 countries were enrolled and observed for 3 years. Eligible participants were heterosexual men aged 16–23 years or men who had sex with men aged 16–26 years.

Furthermore, in the V501-018-11 trial for men and women aged 9–15 years, no breakthrough disease in the forms of genital warts or cervical and/or genital precancers and cancers were observed in the 10-year follow-up period [44].

### 4.3. 9-Valent Vaccine

#### 4.3.1. Young Women

The observation periods for the 9-valent vaccine was 5 years in a Latin American study [52], 6 years in the extension V503-001 [53], 8 years in a Scandinavian study [55] and 8 years in the LTFU extension study [54].

The Latin American study included a randomized, double-blind, controlled trial in women aged 16–26 years and an immunogenicity study in girls and boys aged 9–15 years [52]. The 9-valent vaccine prevented HPV 31/33/45/52/58-related high-grade cervical, vulvar, and vaginal dysplasia with 92.3% effectiveness (95% CI, 54.4–99.6). Antibody responses to the 9-valent HPV vaccine types persisted for more than 5 years.

The V503-001 trial was a randomized, international, multicenter, double-blind study in women aged 16–26 years [61]. In the study, 14,215 participants randomly received the 9-valent vaccine or the 4-valent vaccine on day 1 and at 2 and 6 months. The results showed that the 9-valent vaccine prevented approximately 97% of high-grade cervical, vulvar, and vaginal diseases associated with HPV 31/33/45/52/58, and the increase in antibody titer against HPV 6/11 (LR-HPV) and HPV 16/18 (HR-HPV) was not inferior to that of the 4-valent vaccine. In the extension V503-001 study, the 9-valent vaccine prevented persistent HPV infection, cytological abnormalities, high-grade lesions, and cervical procedures related to the HPV types covered by the vaccine for up to 6 years [53].

In the LTFU extension study of V503-002 [55,56], the 9-valent vaccine elicited HPV antibodies that persisted for at least 7 years after three doses of the vaccine in girls and boys aged 9–15 years of age [54]. Approximately 8 years after vaccination, there were no cases of high-grade CIN, adenocarcinoma-in-situ, vulvar intraepithelial neoplasia, vaginal intraepithelial neoplasia, or genital warts related to the 9-valent vaccine types in women.

In the Scandinavian study, 1448 women aged 16–26 years were followed for the development of HPV16/18/31/33/45/52/58-related high-grade cervical dysplasia [55]. The results suggested that the 9-valent vaccine provides statistically significant protection for at least 6 years and remains effective for 8 years.

#### 4.3.2. Adult Women

To the best of our knowledge, there are no reports on the long-term effects of the 9-valent vaccine in adult women.

#### 4.3.3. Men

In the LTFU extension study of V503-002 [62,63], in approximately 8 years after vaccination, there were no cases of penile intraepithelial neoplasia (PIN) or genital warts in men related to the 9-valent vaccine types [54].

## 5. Long-Term Effectiveness: Real-World Data

There are several reports on bivalent and 4-valent vaccines (Table 2 and Table 3).

### 5.1. Young Women

The observation period of long-term effectiveness for the 4-valent vaccine was 9 years in an Australian study (2015) [49], 10 years in a Danish study [50] and 12 years in another Australian study (2020) [51].

The Australian study (2015) [49] compared HR-HPV prevalence in women aged 18–24 and 25–35 years between the vaccinated and pre-vaccine introduction generations. The results showed that the prevalence of vaccine-targeted HPV types was significantly reduced in the young women 9 years after vaccination.

In the Danish study [50], 19,951 women born in 1993 who received the publicly funded 4-valent HPV vaccine at age 15 (vaccination coverage: 91%) and 15,748 women born in 1983 who were not eligible for publicly funded vaccination (vaccination coverage: <0.1%) were followed for 10 years from age 15 to 25 years of age. The results showed that the relative risk of developing CIN2+ and CIN3+ was significantly lower in women born in 1993 (CIN2+: RR = 0.74 95% CI 0.66–0.82, CIN3+: RR 0.68 95% CI 0.58–0.79).

The Australian study (2020) [51] investigated the effectiveness of the 4-valent vaccine on HPV infection in 1564 women aged 18–35 years (median 24 years). The HPV infection rate of the vaccine target HPV types was 0.7% in the vaccinated group, which was significantly lower than the 5.5% in the unvaccinated group (OR 0.13 95% CI 0.05–0.30) 9–12 years after the vaccine program introduction.

Recently, the long-term 9-year effectiveness of the bivalent vaccine against HPV16/18 and 31/45/52 infection was reported in Japanese women aged 25–26 years [38]. HPV16/18 infection rate was 0% (0/150) in the vaccinated group and 5.4% (15/279) in the unvaccinated group, showing a significant difference (*p* = 0.0018), and the vaccine effectiveness was 100%. Cross-protective-type HPV31/45/52 infection rate in the vaccinated group was significantly lower than that in the unvaccinated group (3.3% vs. 10.0%: *p* = 0.013).

### 5.2. Adult Women

In the Australian study (2015), the 4-valent HPV vaccination program targeting girls aged 12–13 years commenced in 2007, with catch-up vaccination of women aged 14–26 years through 2009. In the study, HPV prevalence in women aged 18–24 and 25–35 years in 2015 was compared with that in 2005–2007 [49]. The results showed that the prevalence of the 4-valent HPV vaccine types decreased from 11.8% (2005–2007) to 1.1% (2015) among women aged 25–35 years (*p* = 0.001), and its effectiveness was confirmed 9 years after vaccination. The authors stated that despite the low vaccination rate of 40.3% among women aged 25–35 years, the significant reduction in HPV prevalence may be due to the effect of strong herd protection.

## 6. Long-Term Persistence of HPV Vaccine-Induced Seropositivity

### 6.1. Bivalent Vaccine

The observation period of long-term persistence of HPV vaccine-induced seropositivity for the bivalent vaccine was 4 years in HPV 032/063 [64], 6 years in Extension NCT00196937 [56], 9.4 years in HPV001/007/023/Extension HPV023 [34], and 10 years in NCT 00,947,115 trial [35].

In the HPV-001 [28], 007 [34], and 023 [33] trials for women aged 15–25 years, the geometric mean titer (GMT) for HPV 16/18 peaked 7 months after the first inoculation and was maintained for 9.4 years (113 months). At that time, GMT was more than 10 times the antibody titer due to natural infection in both HPV 16/18 types [25], and the antibody positive rate was maintained at 100% [34] in the Extension 023 trial.

The P014 trial [29] and its follow-up study (NCT00196937) [30] evaluated the persistence of antibody titers in women aged 15–55 years who received their first vaccination. The study showed that GMTs for HPV 16/18 at 18 months after the first inoculation were in the same range as the plateau GMTs in the HPV-001 and 007 studies. Furthermore, although the GMTs were slightly lower in the age group of 26–55 years than in the age group of 15–25 years, the antibody titer after 48 months was maintained at a higher level than that after natural infection. The antibody titer was maintained for 6 and 10 years after the first inoculation in Extension NCT00196937 [56] and NCT00947115 [35], respectively.

In healthy boys, the P011-NCT00309166 trial examined the efficacy of the bivalent vaccine in healthy boys [37]. The antibody titers of HPV 16/18 in boys aged 10–18 and 10–14 years at 7 months after vaccination were higher than those in women aged 15–25 and 10–14 years in previous studies [57].

### 6.2. 4-Valent Vaccine

The longest period of long-term persistence of HPV vaccine-induced seropositivity for the 4-valent vaccine was 14 years in FUTURE II [45] (P015-21) for women, and 10–10.5 years in the P020-21 [48] and V501-018-11 [44] trials for men.

In the final report of the P015-21 (follow-up study of the P015 FUTURE II) in women aged 16–26 years, the antibody positivity rates of HPV 6/11 (LR-HPV) and HPV 16/18 (HR-HPV) were 90.6%, 91.1%, 98.3%, and 52.4% at 14 years after the first inoculation, respectively [45].

In the P020 trial for men aged 16–26 years, the antibody positivity rates for HPV 6/11/16/18 after 3 years were 86.9%, 78.0%, 92.3%, and 60.7%, respectively [59]. Furthermore, in the follow-up study (P020-21), the antibody positivity rates for HPV 6/11/16/18 were 79.1%, 79.9%, 94.9%, and 40.2% at 10 years after inoculation, respectively [48].

The V501-018-11 [44] trial was conducted on boys and girls aged 9–15 years, and the antibody positivity rates for HPV 6/11/16/18 at 10.5 years after the first inoculation were 86.6%, 87.2%, 94.1% and 59.6% in boys, respectively. In contrast, the antibody positivity rates were 91.0%, 90.1%, 97.7%, and 61.4% in girls, respectively. There seems to be no difference in HPV antibody titers between boys and girls.

### 6.3. 9-Valent Vaccine

The longest period of long-term persistence of HPV vaccine-induced seropositivity for the 9-valent vaccine was 5 years in Extension V503-001 [53] and the Latin American study [52] for young women, and 7 years in the LTFU extension study of V503-002 [54] for girls and boys.

The V503-001 trial confirmed that the antibody response against the 9-valent HPV vaccine type persisted for at least 5 years in women aged 16–26 years, and the antibody positivity rate was ranged from 78% to 100% at 5 years after the third inoculation [53,65]. In the V503-002 trial in girls and boys aged 9–15 years, it was confirmed that the antibody response lasted for at least 5 years after three doses of the 9-valent vaccine [62,63], and the antibody positivity rate against HPV 6/11 (LR-HPV) and HPV 16/18/31/33/45/52/58 (HR-HPV) ranged from 90% to 99% at 5 years after inoculation [62].

A Latin American study [52] confirmed that the antibody titer of the 9-valent vaccine was sustained for 5 years in boys aged 9–15 years.

In the LTFU extension study [54], three doses of the 9-valent vaccine induced HPV antibodies that persisted for at least 7 years in girls and boys aged 9–15 years. GMTs reached its peak at 7 months (1 month after three doses), then declined sharply at 12 months, followed by a slower decrease between 24 and 90 months. Most participants remained seropositive for each 9-valent HPV vaccine type at the last immunogenicity assessment. GMTs at 90 months in the participants were equal to or higher than the GMT at 60 months for women who received the 9-valent vaccine in a previous study [53].

## 7. Conclusions

HPV is mainly transmitted through sexual activity and causes infections that can lead to HPV-associated cancers in both men and women. The preventive effect of HPV vaccines against anogenital and oropharyngeal cancers has been proven in both clinical trials and real-world data. The longest periods of preventive effect for the bivalent, 4-valent, and 9-valent vaccines were 11 years in the Costa Rica trial [36], 14 years in the FUTURE II [45], and 8 years in the LTFU extension study of V503-002 [54] and the Scandinavian study [55], respectively. The sustained clinical effect during the observation period was longest for the 4-valent vaccine. In real-world data, the longest observation period of the vaccine effectiveness was 12 years in an Australian study [51] for the 4-valent vaccine. On the other hand, the longest period of long-term persistence of the HPV vaccine-induced seropositivity for the 4-valent vaccine was 14 years in FUTURE II.

Regarding the bivalent vaccine, it is possible that research on the long-term follow-up trial was not planed due to the launch of the 4-valent and 9-valent vaccines. In some studies of the 9-valent vaccine, the results have not yet been published because of the short observation period [54,55]. The results are expected in the future.

In this review, antibody titers were also mentioned. However, the correlation between the immunological efficacy of antibodies and the clinical efficacy of HPV infection prevention has not yet been established, and it has not been confirmed whether the preventive effect of the vaccine is due to sustained antibody titers. In addition, there is still no consensus on the minimum amount of antibodies needed to prevent HPV infection.

In a national immunization program, most girls and boys are inoculated with HPV vaccine by the time puberty begins; thus, it is important to monitor the vaccine effect at least until the sexually active period in their 20s and 30s.

## Figures and Tables

**Table 1:** Information of available HPV vaccines.

	Bivalent Vaccine Cervarix^®^	4-Valent Vaccine Gardasil^®^	9-Valent Vaccine Gardasil9^®^
Target HPV types	HPV 16/18 (HR-HPV)	HPV 6/11 (LR-HPV) HPV 16/18 (HR-HPV)	HPV 6/11 (LR-HPV) HPV 16/18/31/33/45/52 (HR-HPV)
Schedule	Over 10 years 0, 1, and 6 months	Over 9 years 0, 2, and 6 months	Over 9 years 0, 2, and 6 months
VLP dose	L1 dose 20/20 μg	L1 dose 20/40/40/20 μg	L1 dose 30/30/60/40/20/20/20/20/20 μg
Adjuvant	500 μg aluminum hydroxide, 50 μg 3-O-deacylated-4-monophosphoryl lipid A	225 μg aluminum hydroxyphosphate sulfate	500 μg aluminum hydroxyphosphate sulfate

VLP, virus-like particle; HR-HPV, high-risk HPV type; LR-HPV, low-risk HPV type.

**Table 2:** Long-term effect on bivalent HPV vaccine.

Study	Study Design	Study Subjects	Efficacy and Effectiveness	Follow-Up Period (Seropositivity)	Follow-Up Period (Clinical Effect)
**HPV 001** [28]	Clinical trial	Young women (15–25 years)	Reduced HPV 16/18 infection and HPV 16/18-related cytological abnormalities	3.6 years	3.6 years
**HPV 032/063** [29]	Clinical trial	Young women (20–25 years)	No case of HPV16/18–associated CIN1+	4 years	4 years
**Extension NCT00196937** [30]	Clinical trial	Women (15–55 years)	Sustained anti-HPV-16/18 seropositivity rates	6 years	-
**HPV 007** [31]	Clinical trial	Young women (15–25 years)	No case of persistent infection or CIN2+ associated with HPV-16/18	6.4 years	6.4 years
**VIVIAN study** [32]	Clinical trial	Adult women (> 25 years)	Reduced HPV 16/18 infection, Cytological abnormalities and CIN1+	-	7 years
**HPV 023** [33]	Clinical trial	Young women (15–25 years)	No new infection or CIN2+ associated with HPV 16/18	8.4 years	8.4 years
**Extension HPV023** [34]	Clinical trial	Young women (15–25 years)	No case of HPV16/18 infection and HPV16/18–related Histropathological abnormalities	9.4 years	9.4 years
**NCT 00947115** [35]	Clinical trial	Women (15–55 years)	Sustained anti-HPV-16/18 antibody titers	10 years	-
**Costa Rica Vaccine Tria** [36]	Clinical trial	Young women (18–25 years)	Reduced HPV16/18–related CIN2/3	-	11.1 years
**P011–NCT 00309166** [37]	Clinical trial	Men (10–18 years)	Higher antibody titers of HPV 16/18	7 months	7 months
**Niigata Study** [38]	Real–world data	Young women (25–26 years)	Reduced HPV 16/18 and HPV31/45/52 infection	-	9 years

CIN, cervical intraepithelial neoplasia.

**Table 3:** Long-term effect on 4-valent vaccine.

Study	Study Design	Study Subjects	Efficacy and Effectiveness	Follow-Up Period (Seropositivity)	Follow-Up Period (Clinical Effect)
**FUTURE I** [39]	Clinical trial	Young women (16–24 years)	Reduced HPV-related anogenital disease	2 years	3.6 years
**HPV P007** [40]	Clinical trial	Young women (16–23 years)	No case of HPV 6/11/16/18-related CIN	5 years	5 years
**Extension P007** [41]	Clinical trial	Young women (16–23 years)	Sustained serum anti-HPV 6/11/16/18 immunoglobulin levels	5 years	–
**Extension P019** [42]	Clinical trial	Adult women (24–45 years)	Reduced HPV 6/11/16/18-related CIN	6 years	6 years
**Nordic P015** [43]	Clinical trial	Young women (16–23 years)	No case of HPV 6/11/16/18-related CIN	9 years	8 years
**P019-21** [26]	Clinical trial	Adult women (24–45 years)	No case of HPV 6/11/16/18-related CIN, AIS and EGL	10 years	10 years
**V501-018-11** [44]	Clinical trial	Girls and Boys (9–15 years)	No case breakthrough disease in the form of genital warts or cervical and/or genital precancers and cancers	10.5 years	10 years
**FUTURE II (P015-21)** [45]	Clinical trial	Young women (16–23 years)	No case of HPV16/18 related CIN2+ and cervical cancer	14 years	14 years
**P020-AIN substudy** [46]	Clinical trial	Men-MSM	Reduced AIN (grade 2+)	-	3 years
**NCT00090285** [47] **(Extension P020/P020-11)**	Clinical trial	Men (including MSM) (16–26 years)	No case of HPV 6/11/16/18-related EGL	-	9.5 years in early vaccination 4.7 years in catch-up vaccination
**P020-21** [48]	Clinical trial	Men (16–26 years)	No case of HPV 6/11-related genital warts, HPV 6/11/16/18-related EGL or AIN	10 years	10 years (up to 11.5 years)
**Australian study (2015)** [49]	Real-world data	Women (18–24, 25–35 years)	Reduced vaccine-targeted HPV infection	-	9 years
**Danich study** [50]	Real-world data	Adult women (born in 1993, 1983)	Reduced high-grade CIN	-	10 years
**Australian study (2020)** [51]	Real-world data	Women (18–35 years)	Reduced vaccine-targeted HPV infection	-	12 years

CIN, cervical intraepithelial neoplasia; AIS, adenocarcinoma in situ; AIN, anal intraepithelial neoplasia; MSM, men who have sex with men; EGL, external genital lesion.

**Table 4:** Long-term effect on 9-valent vaccine.

Study	Study Design	Study Subjects	Efficacy and Effectiveness	Follow-Up Period (Seropositivity)	Follow-Up Period (Clinical Effect)
**Latin American study** [52]	Clinical trial	Young women (16–26 years) and girls and boys (9–15 years)	Prevented HPV31/33/45/52/58-related high-grade cervical, vulvar and vaginal dysplasia	5 years	5 years
**Extension V503-001** [53]	Clinical trial	Young women (16–26 years)	Prevented persistent vaccine-targeted HPV infection, cytological abnormalities, high-grade lesions, and cervical procedures	5 years	6 years
**LTFU extension study of V503-002** [54]	Clinical trial	Girls and boys (9–15 years)	No case of vaccine-targeted HPV infection, or high-grade CIN, AIS, VIN, VaIN, PIN, or genital warts	7 years	8 years
**Scandinavian study** [55]	Clinical trial	Young women (16–26 years)	No case of HPV 16/18/31/33/45/52/58-related high-grade CIN	-	8 years

CIN; cervical intraepithelial neoplasia; AIS, adenocarcinoma in situ; VIN, vulvar intraepithelial neoplasia; VaIN, vaginal intraepithelial neoplasia; PIN, penile intraepithelial neoplasia.

## Data Availability

Not applicable.
